# High-Precision Low-Temperature Drift LDO Regulator Tailored for Time-Domain Temperature Sensors

**DOI:** 10.3390/s22041518

**Published:** 2022-02-16

**Authors:** Cristian Răducan, Marius Neag, Alina Grăjdeanu, Marina Țopa, Andrei Negoiță

**Affiliations:** 1 Department of Bases of Electronics, Technical University of Cluj-Napoca, 400114 Cluj-Napoca, Romania; cristian.raducan@bel.utcluj.ro (C.R.); alina.grajdeanu@bel.utcluj.ro (A.G.); marina.topa@bel.utcluj.ro (M.Ț.); 2 Infineon Technologies, 020335 Bucharest, Romania; andrei.negoita@infineon.com

**Keywords:** time-domain temperature sensor, precision LDO, thermal drift, load and line regulation, fast response to load transients, large loop gain, multiple-feedback OpAmp, multiple-feedback stability analysis, ring oscillator

## Abstract

This paper proposes a high-precision LDO with low-temperature drift suitable for sensitive time-domain temperature sensors. Its topology is based on multiple feedback loops and a novel approach to frequency compensation, that allows the LDO to maintain a large DC gain while handling capacitive loads that vary over a wide range. The key design constraints are derived by using a simplified, yet intuitive and effective, small-signal analysis devised for LDOs with multiple feedback loops. Simulation and measurement results are presented for implementation in a standard 130 nm CMOS process: the LDO outputs a stable 1 V voltage, when the input voltage varies between 1.25 V to 1.5 V, the load current between 0 and 100 mA, and the load capacitor between zero and 400 pF. It exhibits a DC load regulation of 1 µV/mA, a 288 µV output offset with a standard deviation of 9.5 mV. A key feature for the envisaged application is the very low thermal drift of the output offset: only 14.4 mV across the temperature range of −40 °C to +150 °C. Overall, the LDO output voltage stays within +/−3.5% of the nominal DC value over the entire line voltage, load, and temperature ranges, without trimming. The LDO requires only 1.4µA quiescent current, yet it provides excellent responses to load transients. The output voltage undershoot and overshoot caused by the load current jumping between 0 and 100 mA in 1 µs are: 10%/22% for CL = 0 and 12%/16% for CL = 400 pF, respectively. A comparative analysis against seven LDOs published in the last decade, designed for similar levels of supply voltage and output voltage and current, shows that the LDO presented here is the best option for supplying sensitive time-domain temperature sensors. The smallest thermal drift of the output offset, smaller than +/−15 mV, that is, 6.7 times smaller than its closest competitor, and the best overall performance when PSR up to 1 kHz, was considered.

## 1. Introduction

Temperature sensors are employed in a wide range of applications, from consumer and industrial to military and aerospace. In general, integrated circuit (IC) sensors provide high linearity and high accuracy, while their size/footprint and complexity remain relatively low [1,2]. This makes them particularly well suited for large systems on chip (SoCs), where they are integrated on the same die as the main circuitry.

IC temperature sensors usually rely on the temperature dependence of the threshold voltage (for CMOS transistors) or the base-emitter voltage (for BJT transistors) to estimate the die temperature. However, for fine CMOS processes operating at low supply voltages, time-domain sensors yield better performance. A recent survey [3] of this trend analyses no less than 23 such sensors reported recently, identifying 12 topologies.

**Figure 1:**
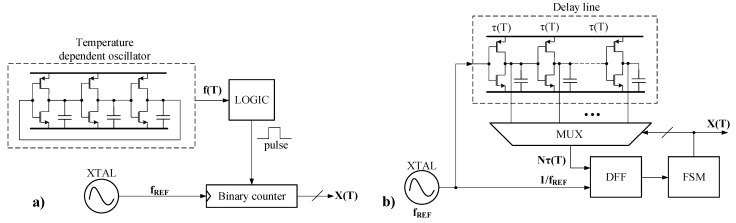
Simplified block diagram of two main time-domain temperature sensor architectures [3]: (**a**) delay time and (**b**) clock period.

Time-domain sensors operate by comparing a temperature-dependent time-related parameter of a signal with a temperature-independent one. Therefore, these sensors can be classified into two broad categories considering the signal parameter they exploit: delay time and clock period [3]. Figure 1 presents the block diagrams for these sensor categories.

The temperature-dependent oscillator shown in Figure 1a is usually implemented by a ring oscillator. These circuits are particularly sensitive to variations of the supply voltage. A similar chain of CMOS inverters is used to implement the delay line used by the clock-period-based architecture shown in Figure 1b. It follows that both architectures for time-domain temperature sensors are sensitive to variations of the supply voltage. Not surprisingly, implementations of such sensors published recently reported supply sensitivities of up to 1600 °C/V, leading to temperature errors of up to +/−4 °C [3].

This paper focuses on providing a supply unit tailored for time-domain temperature sensors, that will allow them to substantially reduce the error caused by variations of their main supply line, inevitable within an integrated circuit. The envisaged low-dropout voltage regulator (LDO) should provide a stable and accurate voltage, whose deviation from the ideal value must have a very small thermal drift over the entire temperature range of the sensor. Another design challenge is to ensure fast responses to load and line transients without the help of an external decoupling capacitor, which would increase the number of required pins. In particular, one has to minimize the voltage undershoot/overshoot caused by the large load current spikes generated by the switching of the ring oscillator and/or the auxiliary digital circuitry. The LDO power consumption has to be very low, suitable for battery-powered smart systems.

A large gain in the LDO voltage-control loop is required to ensure good accuracy and DC line and load regulations. To achieve this in smaller technology nodes one needs to employ multiple gain stages, which in turn makes frequency compensation more difficult. Not surprisingly, some of the LDOs with fast responses to load transients sacrifice precision for speed. A popular error amplifier (EA) employed in fast LDO designs for its excellent dynamic behavior is the class-AB common-gate EA proposed in [4]. The topology was further improved by employing recycling techniques [5,6,7,8,9,10], local common-mode feedback [7,10,11,12,13], and by a more efficient frequency compensation circuit [10]. Despite its speed, the class-AB input EA is not a suitable candidate for the application envisaged here due to its large offset voltage, caused by inherent mismatches within the input stage. Furthermore, it cannot provide the large gain required for the voltage control loop on its own.

To sidestep the trade-off between transient performance and power consumption, some LDO designs employ adaptive biasing for the error amplifier. The LDO reported in [14] employs continuous adaptive biasing obtaining good transient performance. However, it does not achieve the lowest power consumption since the quiescent current increases with the load current by two orders of magnitude. By contrast, the adaptive biasing employed in [15] is activated only during the transient variations of the output voltage. This approach does not yield the best transient performance but it allows the LDO to maintain a low power consumption even at large load currents.

These limitations can be surpassed by using LDOs with multiple feedback loops such as those proposed in [16,17,18,19]. Stability analysis of circuits with one feedback loop is well covered by the classical feedback theory and several methods are available for deriving the phase- and gain-margins associated with the circuit return ratio [20,21,22]. Extending this approach to the analysis of circuits with multiple feedback loops is not straightforward. The return ratio of such circuits usually has a complex mathematical expression, a ratio of high-order polynomials. One can try to simplify it by using approximations and algebraic methods [23] but such convoluted analysis provides no insight into the circuit operation, nor its design trade-offs. Instead, the simpler, yet more intuitive stability analysis introduced in [10] was further developed to suit the circuit proposed in this work.

Section 2 of this paper introduces a multiple feedback loop LDO suitable to time-domain temperature sensors. The main idea is to use a fast LDO core to ensure fast responses to the line and load transients, then overcome the trade-off between speed and precision specific to that core by closing an additional, high gain feedback loop around it. The next section presents a design example of an LDO implemented in a standard 130 nm CMOS process. Simulation results and measurements performed on the test chip validate the design. The final section presents a comprehensive comparison with seven similar LDOs, as well as the main conclusions drawn from this work.

## 2. Proposed Fast LDO with Multiple Feedback Loops

### 2.1. Proposed Schematic

**Figure 2:**
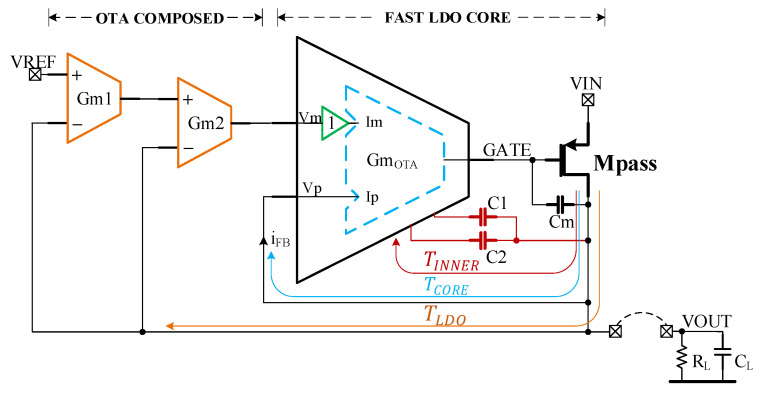
Topology of the proposed LDO: three Gm cells, each of them connected to the LDO output by total negative feedback loops. Each cell can be optimized individually, for different features.

The topology of the LDO proposed here is shown in Figure 2. It consists of three Gm cells, each of them connected to the LDO output by total negative feedback loops. This arrangement allows for each Gm cell to be optimized individually, targeting different features. The innermost cell—denoted $Gm_{OTA}$ in Figure 2—together with the pass transistor compose the fast core of the LDO. The $Gm_{OTA}$ cell drives the large parasitic capacitance present at the pass transistor gate, so it is optimized for high speed and wide bandwidth. The other two cells—denoted Gm1 and Gm2 in Figure 2– create a composite OTA (COTA) that helps enlarge the DC gain of the voltage-control loop of the LDO and determines its input offset voltage. Therefore, the COTA is optimized for large gain and low offset.

**Figure 3:**
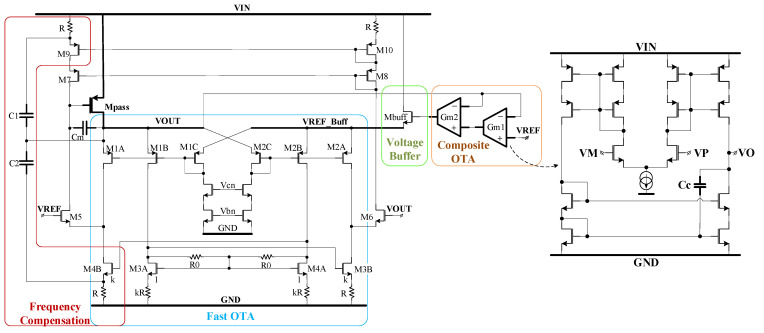
Transistor-level schematic of the proposed fast LDO with multiple feedback loops.

A transistor-level implementation of the LDO is shown in Figure 3. The $Gm_{OTA}$ cell of Figure 2 is composed of the common-gate transconductor denoted “Fast OTA”, an output stage based on cascaded current mirrors and a voltage buffer on the VREF input. This circuit is similar to the one introduced in [10], with the main exception being the replacement of the closed-loop voltage buffer in [10] with the source follower Mbuff. 

The source follower does not significantly impact the frequency characteristics of the LDO loop gain. Thus, it is possible to ensure the stability of the LDO presented here, even if it comprises one more stage than the LDO reported in [10]: the additional gain stage implemented by the composite OTA, which brings in another two poles and one zero.

The “FAST LDO CORE” of Figure 2 is implemented by the circuits denoted ”Fast OTA” and ”Frequency Compensation” in Figure 3. The current recycling and local common-mode feedback techniques for improving the slew rate detailed in [10] are employed here, as well:

(1) The current recycling introduced in [24], realized here by using two transistors for each input (M1A_B and M2A_B) and the current mirrors M3A-M3B and M4A-M4B.
(2) The local common-mode feedback (LCMFB) introduced in [25], realized here by the resistors R0, helps to further increase both the gain and the slew rate.

The small-value capacitor, $C_{m}$, connected between the gate and the drain of Mpass, helps speed up the initial phase of the LDO response to output voltage variations, when the current generated by the class-AB Fast OTA and capacitors $C_{1}$ and $C_{2}$ is rather small. This section of the LDO ensures the required fast responses to line and load transients, but does not provide a large DC gain.

The additional gain stage realized by the COTA, implemented by the Gm cells denoted Gm1 and Gm2, helps achieve the large DC gain necessary for meeting the required accuracy in DC. Such a gain stage could have been implemented by using only one OTA, but employing two OTAs connected in a cascade, as shown in Figure 3, brings two advantages compared with the single-OTA stage:

(i) A larger DC gain for the LDO;
(ii) A feed-forward signal path is created by having the negative inputs of both Gm1 and Gm2 connected to the LDO output. This is a key feature for obtaining a suitable Phase Margin for the LDO, as it will be shown in Section 2.2.

Both Gm1 and Gm2 employ a symmetrical OTA structure with the NMOS input transistors and Miller-type frequency compensation. Degenerated PMOS current mirrors and a cascaded NMOS current mirror were used to ensure the required large DC gain. More importantly, the input differential stage can be optimized for low offset, without undue constraints regarding its speed.

### 2.2. Stability Analysis

The LDO proposed in Figure 3 comprises three feedback loops which are highlighted in the circuit topology shown Figure 2:

- The inner loop—whose gain is denoted $T_{INNER}$ in Figure 2—is closed around the Fast OTA by the frequency compensation circuit based on capacitors C1 and C2;
- The total feedback loop closed around the FAST LDO CORE forms the core feedback loop—whose gain is denoted $T_{CORE}$ in Figure 2;
- The outer loop—whose gain is denoted $T_{LDO}$ in Figure 2—is the main voltage control loop of the LDO; it combines the two direct connections between the LDO output and the inverting inputs of the two transconductors within the COTA, Gm1, and Gm2.

The LDO stability depends on all three loops: the LDO is stable when the loop gain of each of them meet the general stability criteria [10]:

(1) $$T_{INNER}\neq \;1\;\;\;\&\;\;\;T_{CORE}\neq \;1\;\;\;\&\;\;\;T_{LDO}\neq \;1\;$$

Brute force circuit analysis of the small-signal equivalent of the circuit shown in Figure 3 yields complex, high-order expression for these loop gains, which are awkward to use by the circuit designer. Therefore, the conventional approach is to reduce it to a more manageable equivalent expression by using a series of approximations, which in turn, are valid only if several sizing conditions are imposed. This algebra-driven approach is not conducive to an intuitive understanding of the design constraints and the sizing equations required by a circuit designer.

Instead, let us extend the approximate, yet effective and intuitive, stability analysis method introduced in [10] to the multiple-loop LDO proposed here. Its main points are:

- First, the multiple-loop topology can be simplified iteratively, starting from the inner loop and moving outwards. At each step, the inner-most feedback section is replaced by its closed loop equivalent yielded by using classical feedback theory, thus simplifying the analysis of the entire circuit.
- The loop gain of each “*Tx* loop” is derived by using the Rosenstark theorem [21]:
  (2) $$T_{x}=\frac{T_{x}^{I}T_{x}^{V}}{T_{x}^{I}+T_{x}^{V}}$$
- Note that the voltage and current transfer ratios appear “in parallel”; this suggests that, if one of these ratios is far smaller than the other one, the resulting loop gain is mainly determined by the smaller transfer ratio.

For example, if the loop comprises a high-impedance point that can be used as the loop-breaking-point, the resulting voltage transfer ratio will provide a good approximation for the entire loop gain. Thus, we only need to compute both transfer ratios only when no high-impedance point for breaking the loop is available. However, even in such cases, one of the transfer ratios can dominate the loop gain in the relatively narrow frequency range around the unity-gain frequency of Tx we are interested in.

**Figure 4:**
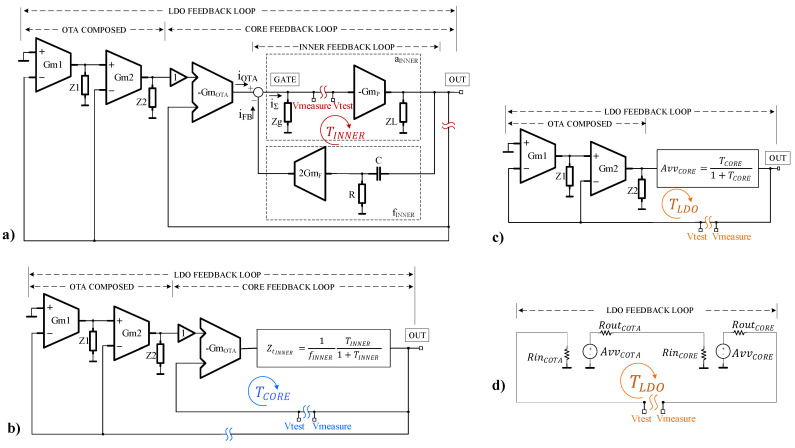
Small signal diagram of the LDO showing simplified representations of the small-signal model for computing $T_{INNER}$ (**a**), $T_{CORE}$ (**b**), and $T_{LDO}$ (**c**,**d**) feedback loops. After each step, the resulting loop gain is replaced by its equivalent circuit, derived by using classical feedback theory.

Let us begin from the inner-most feedback loop shown in Figure 4a. As detailed in [10], it can be replaced by its closed-loop equivalent, that is, a current-input, voltage-output block with the transfer function:

(3) $$Z_{t_{INNER}}=\frac{v_{out\;}}{i_{\mathrm{OTA}}}≅\frac{1}{f_{INNER\;}}\frac{T_{INNER\;}}{1+T_{INNER\;}}$$

for which:

(4) $$T_{INNER\;}≅T_{INNER}^{V}=\frac{v_{measure}}{v_{test}}=\frac{2sRC\left(G_{mF}G_{mP}R_{g}R_{L}\right)}{\left(1+sR_{g}C_{g}\right)\left(1+sR_{L}C_{L}\right)\left(1+sRC\right)}$$

(5) $$a_{INNER\;}=\frac{G_{mP}R_{g}R_{L}}{\left(1+sR_{g}C_{g}\right)\left(1+sR_{L}C_{L}\right)}\;$$

(6) $$f_{INNER\;}=2\frac{sRCG_{mF}}{\left(1+sRC\right)}\;$$

where C = C1 = C2, $v_{test}$ represents the test voltage applied to the loop after breaking it at the point indicated in Figure 4a, and $v_{measure}$ is the voltage outputted by the loop there.

$T_{INNER}$ has the same expression as its loop gain with the same name in [10]. The detailed analysis presented there yielded two main design constraints for the R and C comprising the frequency compensation network, valid here, as well:

(7) $$max\left\{\frac{C_{L}}{G_{mF}G_{mP}R_{g}},\frac{C_{g}}{G_{mF}G_{mP}R_{L}}\right\}\leq RC\leq \sqrt{\frac{C_{g}C_{L}}{G_{mF}G_{mP}}}\;$$

By replacing the LDO inner feedback loop with its closed-loop equivalent (3), one obtains the LDO model shown in Figure 4b. This is also a multiple-loop circuit, with the loop closed around the LDO fast core being the inner-most loop in this case. A second topological transformation is necessary to replace the entire section denoted by “CORE FEEDBACK LOOP” in Figure 4b with its equivalent closed-loop circuit, shown in Figure 4c. The core feedback loop has a series-parallel topology, with unitary feedback transmittance. Therefore, the equivalent closed-loop circuit has the voltage–voltage gain $Avv_{CORE}=\frac{T_{CORE}}{1+T_{CORE}}$, while its input and output impedances are obtained by multiplying, respectively dividing, by the factor ($1+T_{CORE\;}$), the input/output impedances of the open-loop circuit. The $T_{CORE}$ loop gain can be derived by using (2), where the voltage and the current transfer ratios are:

(8) $$T_{CORE}^{V}=\frac{v_{measure}}{v_{test}}=G_{mOTA\;}Z_{t\_INNER\;};\;T_{CORE}^{I}=\frac{i_{measure}}{i_{test}}≅\left(1+g_{m3,4B}R_{g3,4B}\right)R_{g}G_{mP}\frac{1}{1+sR_{g}C_{g}}\;$$

To analyze the condition $T_{CORE}\neq$ 1, one can use the results obtained in [10], as this section of the circuit proposed in Figure 3 is similar to the entire circuit reported in [10]: near the unity gain frequency $T_{CORE}^{V}\ll T_{CORE}^{I}$, so the gain- and phase-margin corresponding to $T_{CORE}$ can be derived by analyzing the frequency characteristics of $T_{CORE}^{V}$. This way, one reaches the conclusion that the following design constraint must be observed in order to ensure a good phase margin for the $T_{CORE}$:

(9) $$\frac{G_{mOTA\;}}{G_{mF\;}}<1\;$$

Figure 4c presents the small-signal model of the LDO obtained after the second topological transformation. It can be further simplified by replacing the composite with its standard two-port equivalent, as shown in Figure 4d. This way, the small-signal model of the LDO was reduced from the rather complex multiple-loop representation shown in Figure 4a to the single-loop circuit shown in Figure 4d. The latter allows for a much-simplified derivation of the LDO loop gain, $T_{LDO}$.

The voltage gain of the equivalent model of the composite OTA shown in Figure 4d can be expressed as follows:

(10) $$Avv_{COTA}=-\left[\left(\frac{A1}{1+\frac{s}{\omega_{p1}^{COTA}}}+1\right)\cdot \frac{A2}{1+\frac{s}{\omega_{p2}^{COTA}}}+1\right]$$

where A1, $\omega_{p1}^{COTA}$, and A2, $\omega_{p2}^{COTA}$ are the DC gain and dominant pole of Gm1 and Gm2, respectively.

The series-parallel feedback topology of the core feedback loop ensures that $Rin_{CORE}\gg Rout_{COTA}$ and $Rin_{COTA}\gg Rout_{CORE}$. Therefore, $T_{LDO}$ is mainly determined by its voltage transfer ratio component, $T_{LDO}^{V}$, which can be approximated as follows:

(11) $$T_{LDO}≅T_{LDO}^{V}=\frac{v_{measure}}{v_{test}}≅Avv_{COTA}Avv_{CORE}$$

The advantage of employing this topology for the two-stage composite OTA is the presence of the internal feedforward path that bypasses Gm1. Unlike conventional two stage OTAs, the feedforward path of the composite OTA introduces a real zero in the expression of $T_{LDO}$; by combining (10) and (11) one obtains:

(12) $$T_{LDO}=Avv_{COTA}Avv_{CORE}=\left(A1A2+A1\right)A_{CORE}\;\frac{\left[1+\frac{s}{\omega_{p1}^{COTA}\left(1+A1\right)}\right]}{\left(1+\frac{s}{\omega_{p1}^{COTA}}\right)\left(1+\frac{s}{\omega_{p2}^{COTA}}\right)\left(1+\frac{s}{\omega_{u}^{CORE}}\right)}$$

$Avv_{COTA}$ exhibits two real poles, placed at the angular frequencies, $\omega_{p1}^{COTA}$ and $\omega_{p2}^{COTA}$, and one zero given by:

(13) $$\omega_{z}^{\mathrm{COT}A}=\omega_{p1}^{COTA}\left(1+A1\right)$$

A sizing strategy can be devised whereby the feed-forward zero provides a very useful boost to the $T_{LDO}$ phase margin. This idea will be exploited in the Section 3.

## 3. Design Example

### 3.1. LDO Requirements and Design Strategy

The LDO proposed in Figure 3 was implemented in a standard 130 nm CMOS process with requirements tailored for supplying several time-domain temperature sensors with architectures similar to the ones in [26,27,28,29,30,31,32], as well as their additional digital processing and control circuitry.

First, the LDO should maintain the DC level of the output voltage within +/−3.5% of its nominal value of Vout = 1 V, over the entire range of input voltages (from 1.25 V to 1.5 V), load currents (0 to 100 mA), and temperature (from −40 °C to +150 °C), without trimming. Assuming a straightforward trimming of the output voltage—by simply adjusting the LDO reference voltage at room temperature and nominal supply and load—the post-trim thermal drift of the LDO output voltage must be smaller than +/−15 mV across the full temperature range.

Most time-domain temperature sensors employ conversion rates between 1 kHz and 10 kHz [3], to avoid self-heating effects which may degrade the sensor performance [33]. Therefore, the PSR performance of the LDO should be optimized for these frequencies: at least 80 dB up to 1 kHz and 40 dB at 10 kHz.

In order to accommodate several types of sensors and their additional support and control digital circuitry, the LDO must handle a fairly wide range of load capacitances, CL, from practically 0 to 400 pF, and effectively no ESR.

The LDO response to line and load transients should maintain the output voltage overshoot and undershoot (+/− ΔVout @ ΔVIN, ΔIL) within +/−20% of the nominal Vout value—the typical requirement for SoCs.

An aggressive target was set for the LDO power consumption: no more than 1.5 uA quiescent current. This ruled out most of the previously reported LDOs, with only a few notable exceptions, such as [10]. However, the LDO introduced in [10] cannot provide the DC accuracy required here.

Besides the obvious requirement to ensure stability for all operating conditions, the LDO envisaged here should exhibit 40 degrees of phase margin for CL = 0 and maximum load current. Some design leeway is provided by setting to 15 degrees the minimum value of the LDO phase margin that should be maintained over the entire range of load current and capacitance, as well as the temperature set above. This value is smaller than the typical target for LDOs, that is 25 to 30 degrees, but one should note that stable LDOs with a phase margin below10 degrees have been reported recently [10].

**Figure 5:**
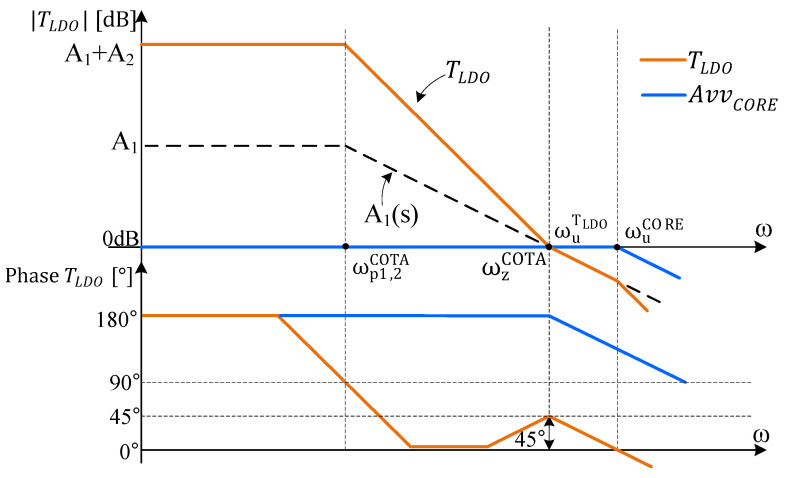
Module (**top**) and phase (**bottom**) frequency characteristics of the $T_{LDO}$ described by (12). $T_{LDO}$ represents an inverting gain; therefore, its phase characteristics start from 180°.

The frequency compensation strategy follows the previous analysis which showed that $T_{LDO}$ exhibits three poles and only one zero. From (13), one notices that the position of the zero is dependent on the position of $\omega_{p1}^{COTA}$ multiplied by a constant factor, A1. This reduces the degree of freedom the designer has in placing the zero. Therefore, to ensure a suitable phase margin for $T_{LDO}$, the position of the poles and zeroes should follow the placement shown in Figure 5.

First, the zero of $Avv_{COTA}$ ($\omega_{z}^{COTA})$ must occur before the unity gain frequency, $\omega_{u}^{CORE}$, of $Avv_{CORE}$:

(14) $$\omega_{z}^{COTA}<\frac{1}{10}\omega_{u}^{CORE}=\frac{1}{10}\frac{G_{mOTA\;}}{2RCG_{mF\;}}$$

Second, the zero $Avv_{COTA}$ must occur at the 0 dB crossover of $T_{LDO}$, in order to push the phase margin up to 45°. This is achieved by forcing $\omega_{p2}^{OTA}$ to equal $\omega_{p1}^{OTA}$. Therefore, one can employ the same OTA circuit for Gm1 and Gm2.

One notices that for angular frequencies larger than $\omega_{p1}^{OTA}$ and $\omega_{p2}^{OTA}$, the $T_{LDO}$ phase characteristic drops close to zero degrees, before rising back up to 45°. Conventional designs avoid such loop-gain characteristics, out of concern that particular parasitic capacitances or process–voltage–temperature (PVT) operating conditions will somehow contrive to push the $T_{LDO}$ phase below zero degrees before $\omega_{u}^{LDO}$, a situation commonly—yet mistakenly—associated with unstable systems.

However, such concerns are unwarranted in this case for two reasons. First, there is no room in this circuit for an unaccounted for parasitic capacitance large enough to generate another pole at such a low frequency. Second, even if the phase were to drop below zero, the circuit remains stable as long as the $T_{LDO}$ phase characteristic gets back to positive values before the unity gain frequency [34]. Note that the Bode stability criterion is not suitable for analyzing conditionally stable circuits and that the Nyquist criterion should be used instead.

Equation (12) indicates the need for the $Avv_{CORE}$ to have a wide bandwidth, that is, for the unity gain angular frequency, $\omega_{u}^{CORE}$, to have a large value.

Here, is a seven-step design strategy based on the approach described above:

(S1) Size the pass transistor by using a simple model for the error amplifier that includes only the DC gain and the output impedance.
(S2) Design the fast LDO core focusing on getting the largest possible value for $\omega_{u}^{CORE}$, within the current consumption budget.

Based on the LDO requirements above, the fast LDO core was designed following the steps and 3D representations detailed in [10]. This yielded the following values for the frequency compensation network, R0 and current mirror gain “k”: C1 = C2 = 15 pF, R = 20 kΩ, R0 = 300 kΩ, and k = 2. The resulting $\omega_{u}^{CORE}$ had the minimum value of 233 krad/s for ILmin and CLmax.

(S3) Use (14) to compute $\omega_{z}^{COTA}$ considering the worst-case value for $\omega_{u}^{CORE}$ obtained in the previous step. In our case this approach yielded $\omega_{z}^{COTA}$= 111 krad/s.
(S4) Derive the required $T_{LDO}$ DC gain from the LDO requirements, then split it between the gain stages implemented by Gm1 and Gm2.

The output voltage deviation from the nominal DC value is caused by variations of the supply voltage, load current, and temperature. The error budget of +/−3.5% had to be split between line regulation, load regulation, offset voltage, and temperature drift. In general, a large DC value of the loop gain—well over 120 dB—ensures that variations of the load current have no significant impact on the DC value of Vout [19]. Therefore, we set the target DC gain value of $T_{LDO}$ to 140 dB. This value was split equally between the gain stages implemented by Gm1 and Gm2, resulting in 70 dB of gain for each of them.

(S5) Use (13) to compute the value of $\omega_{p1}^{COTA}$.
  In our case, this yielded $\omega_{p1}^{COTA}$= 27 rad/s.
(S6) From S4 and S5 compute the required compensation capacitor Cc. Note that this value is to be used in both Gm1 and Gm2.
  In our case, the required capacitor value was Cc = 6 pF.
(S7) Complete the design by sizing the transistors and resistors within the circuit. Due to the modular architecture of the LDO, the composite OTA can be optimized for low offset and temperature drift, independently of the fast LDO core, without impacting the transient response. For example, transistors with large widths and lengths were used to implement the input stages of both Gm1 and Gm2 cells in Figure 3. The remaining current budget was split equally between Gm1 and Gm2.
(S8) Optimize design considering Monte Carlo and PVT simulations; in particular, find a suitable value for capacitor Cm that helps improve the initial phase of the LDO response to load transients. In our case, the optimum Cm value was found to be 4 pF.

### 3.2. Simulation Results

**Figure 6:**
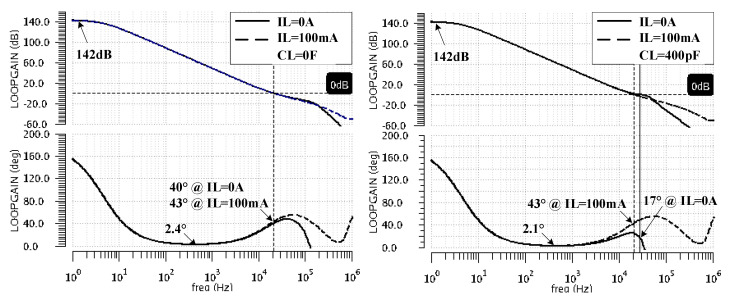
The frequency characteristics of the LDO loop gain, $T_{LDO}$, for CL = 0 F (**left**) and 400 pF (**right**) and extreme IL values: 0 mA and 100 mA.

Figure 6 shows the frequency characteristics of the LDO loop gain, $T_{LDO},$ for the extreme values of the load capacitance (CL = 0 and 400 pF) and load current (IL = 0 and 100 mA). One notices that the DC gain of 142 dB is maintained regardless of the load current. As expected, the phase characteristics drop to values near zero (due to double pole introduced by the COTA, $\omega_{p1,2}^{OTA}$), then rise (due to $\omega_{z}^{COTA}$ of the COTA) before the unity gain frequency. For CL = 0, the resulting phase margin values obtained for $T_{LDO}$ are about 20° for all IL values. For the maximum CL of 400 pF, the unity gain angular frequency, the fast LDO core, $\omega_{u}^{CORE}$, is smaller at zero load current than at high load currents. This explains the different phase margin values obtained for $T_{LDO}$: from 43 degrees at maximum load current, it drops to 17° for IL = 0 A. The LDO remains stable but one may think that the phase characteristics get too close to zero for comfort; if so, one should reduce the DC gain, which will reduce the distance between the double pole located at $\omega_{p1,2}^{OTA}$ and the zero located at $\omega_{z}^{COTA}$—see Equation (13).

**Figure 7:**
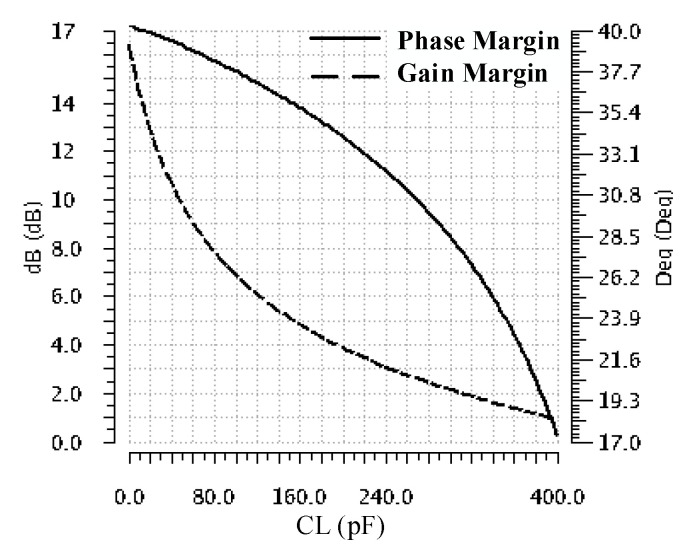
The phase and gain margin variation with CL ranging from zero to 400 pF at IL = 0 A.

Figure 7 presents the variation of $T_{LDO}$ phase and gain margins with the CL value for the no-load-current operation. It indicates that, as the value of the load capacitor increases, the phase margin monotonically decreases, reaching a minimum of 17° for CL = 400 pF. This confirms the theoretical analysis made in Section 2.2 and Section 3.1. Although the phase margin is maintained above 15°, as required, the gain margin drops below 10 dB for CL values above 200 pF. Consequently, for larger values of CL, one should expect increased ringing on the LDO response to load jumps.

**Figure 8:**
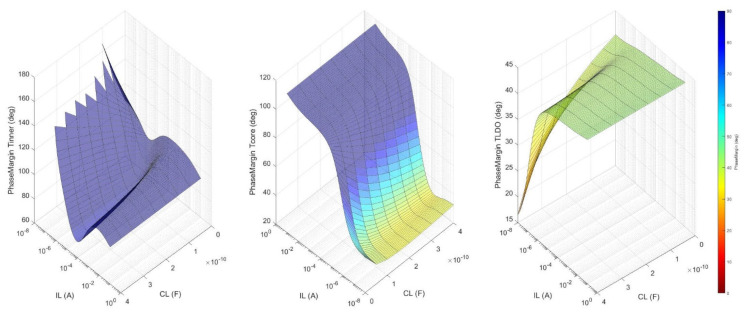
The phase margin for $T_{INNER},\;T_{CORE}$, and $T_{LDO}$ when CL and IL are swept over their entire range of values: CL = 0 to 400 pF and IL= 0 to 100 mA.

To make sure the LDO is stable one has to check that the circuit meets the requirements set by (1) for all operating conditions. This means verifying the phase and gain margins for each of the three loop gains depicted in Figure 4—$T_{INNER},\;T_{CORE}$, and $T_{LDO}$—when CL and IL are swept over their entire range of values—CL = 0 to 400 pF, IL = 0 to 100 mA—as shown in Figure 8. One notices that each loop exhibits a suitable phase margin for all CL and IL values: $PM_{T_{INNER}}$ > 60°, $PM_{T_{CORE}}$ > 20°, and $PM_{T_{LDO}}$ > 15°.

**Figure 9:**
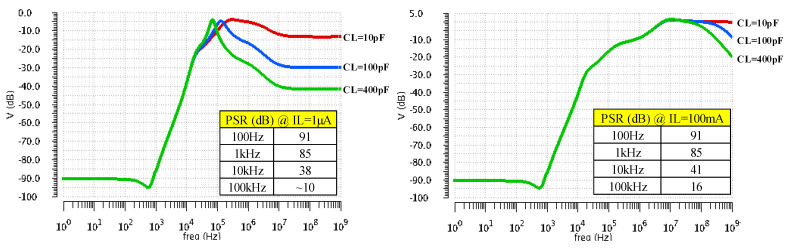
LDO PSR simulation results for three CL values (10 pF, 100 pF, and 400 pF) at extreme values for IL: 1 µA and 100 mA.

Figure 9 shows the LDO PSR frequency characteristics for three CL values (10 pF, 100 pF, and 400 pF) at extreme values for IL (1 µA and 100 mA). One notices that at up to 1 kHz the LDO exhibits a PSR of at least 85 dB regardless of loading conditions. At 10 kHz, the PSR drops to about 40 dB.

**Figure 10:**
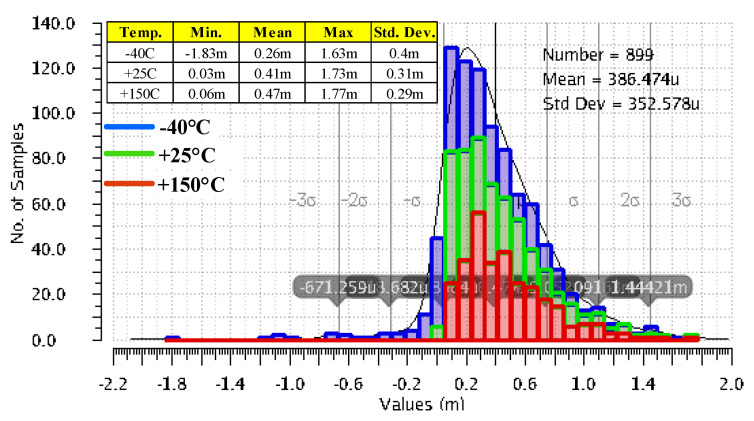
Variation of the LDO output voltage when VIN changes its value from 1.25 V to 1.5 V, while the load current is kept constant, IL = 1 mA. Results yielded by 300 Monte Carlo simulation runs for three temperatures, −40 °C, +25 °C, and +150 °C.

Figure 10 presents the variation of the LDO output voltage when VIN changes its value from 1.25 V to 1.5 V, while the load is kept constant, IL = 1 mA. These results were yielded by 300 Monte Carlo simulation runs for each of room and extreme temperature. The variation is remarkably small in most cases, except for a few outliers at minimum temperature and input voltage, that yield the minimum value of −1.83 mV. At VIN = 1.25 V, the gate voltage necessary to drive the voltage buffer, Mbuffer, in Figure 3 gets close to its minimum required value for proper operation; moreover, at −40 °C, the threshold voltage of the Mbuffer reaches its maximum value.

**Figure 11:**
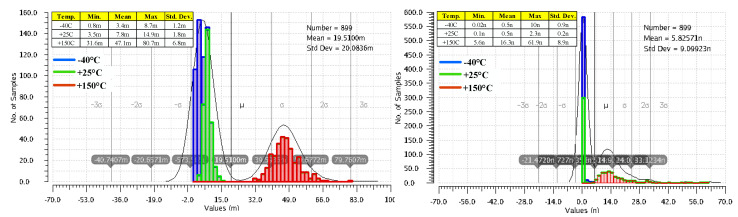
Variation of the LDO output voltage when IL changes its value from 0 A to 100 mA, for the LDO reported in [10] (**left**) and the proposed LDO, shown in Figure 3 (**right**). Results yielded by 300 Monte Carlo simulation runs for three temperatures, −40 °C, +25 °C, and +150 °C, and constant line voltage, VIN = 1.5 V.

Figure 11 presents side-by-side the variation of the LDO output voltage when IL changes its value from 0 A to 100 mA, for the LDO reported in [10] and for the LDO proposed here, shown in Figure 3. These results were yielded by 300 Monte Carlo simulation runs for each of room and extreme temperatures, while the input voltage was kept constant, VIN = 1.5 V. As expected, the very large DC gain of the LDO described here helps improve its load regulation: the maximum output voltage variation is 61.9 nV, six orders of magnitude smaller than the one obtained for the LDO in [10], 80.7 mV. As explained in Section 2, the main difference between the two LDOs is the additional gain stages implemented by the composite OTA for the proposed LDO, shown in Figure 3.

**Figure 12:**
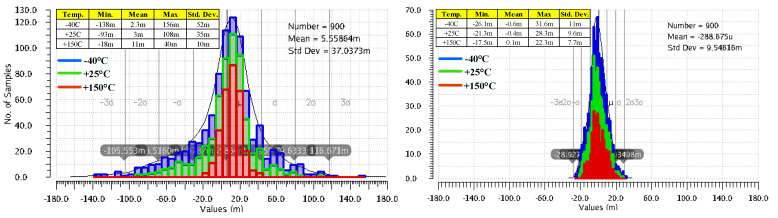
Deviation caused by component mismatches of the LDO output voltage from its nominal DC value for the LDO reported in [10] (**left**) and the proposed LDO, shown in Figure 3 (**right**). Results of 300 Monte Carlo simulation runs for three temperatures, −40 °C, +25 °C, and +150 °C, and constant line voltage and loading: VIN = 1.5 V, IL = 1 mA, and CL = 100 pF.

Figure 12 presents side-by-side the deviation caused by component mismatches of the output voltage from its nominal DC value, V_OS_, for the two LDOs mentioned above. They were yielded by 300 Monte Carlo simulation runs for each of room and extreme temperatures, with VIN set to 1.5 V and IL = 1 mA. The common gate error amplifier used in [10] is fast but exhibits a large output voltage offset: the mean value is 5.5 mV and the standard deviation is very large at 37 mV. The composite OTA added to the LDO shown in Figure 3 ensures a significantly better accuracy: a mean value of 288 µV and a standard deviation of only 9.5 mV, that is, 3.8 times smaller than the LDO reported in [10].

**Figure 13:**
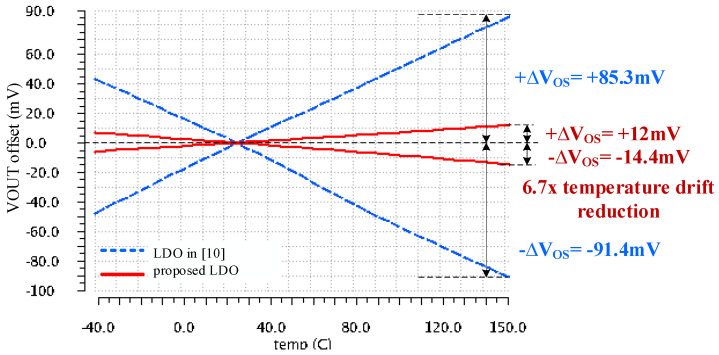
Variation with temperature of the worst-case output voltage errors for the LDO reported in [10] (dotted blue lines) and the LDO proposed here, shown in Figure 3 (continuous red lines). For both LDOs, the operating conditions were VIN = 1.5 V, IL = 1 mA, and CL = 100 pF, and the output voltage was trimmed by adjusting the LDO reference so that Vout = 1 V at +25 °C.

Even more important for LDOs required to supply time-domain temperature sensors is the temperature drift of the output voltage error. Figure 13 presents the V_OS_ variation with temperature for the LDO reported in [10] and the LDO described in this work. The characteristics shown in Figure 13 were obtained in three steps:

- The Vos variation with temperature was monitored over 300 Monte Carlo runs of DC temperature sweeps.

- The worst-case runs that yielded the largest differences between the minimum and maximum Vos values over temperature, were identified.

- The corresponding characteristics were shifted by modifying the LDO reference voltage, so that the output voltage reached its nominal value at +25 °C, that is Vos = 0.

Figure 13 shows that the output voltage error of the LDO reported in [10] exhibits a fairly large post-trim thermal drift, 176.7 mVpkpk. The corresponding value for the LDO proposed here is almost seven times smaller, of only 26.4 mVpkpk.

Results presented in Figure 10, Figure 11, Figure 12 and Figure 13 demonstrate that for the proposed LDO, the output voltage error caused by component mismatches and variations of the line voltage, load current, and temperature, ΔVout_DC, remains within +/−35 mV without trimming and within +/−15 mV after trimming, thus meeting the requirements set out in Section 3.1.

**Figure 14:**
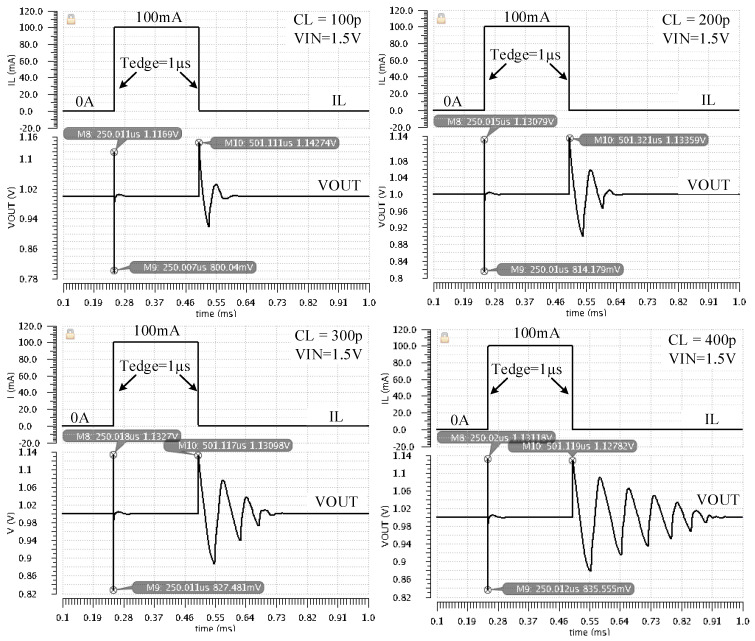
Simulated LDO response to load current steps for four values of CL and VDD = 1.5 V. The load current jumps from IL = 0 A to ILmax = 100 mA (at t = 250 µs) and back to zero (at t = 500 µs) in 1 µs.

Figure 14 presents the LDO responses to positive and negative load current steps, with IL jumping between its minimum (IL = 0) and maximum (IL = 100 mA) values in 1 µs, for load capacitor values between 100 pF and 400 pF. As the CL value increases, so does the time it takes the LDO output to settle after a negative load step, with IL jumping down from 100 mA to zero. These results are in line with the ones obtained by AC simulations: the gain and phase margin decrease with larger values of CL. However, for the envisage application the longer settling time is not an issue.

### 3.3. Simulation Results for the Temperature-Dependent Oscillator Supplied by the Proposed and Reference LDOs

**Figure 15:**
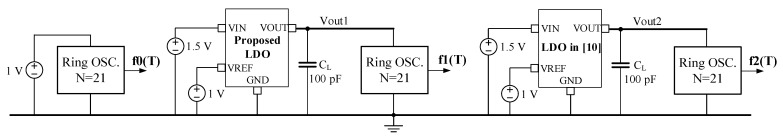
The testbench circuit employed to analyze the impact various supply sources have on the temperature-dependent ring oscillator.

A time-domain temperature sensor was designed, based on the temperature-dependent ring oscillator architecture shown in Figure 15. The oscillator comprised 21 delay stages, each of them consisting of tailored-sized CMOS inverters loaded by placed capacitors with a small variation over temperature. Its nominal frequency when operating at the temperature (T) of 25 °C was set to 10 kHz.

This section presents data on the impact the circuit that provides the supply voltage has on the sensor accuracy. The sensor consists of a sensing core—the ring oscillator—and digital signal processing circuitry. The former is sensitive to variation of its supply line, while the latter is practically insensitive to both temperature and supply voltage. Therefore, the analysis should focus on the impact the supply source has on the oscillator frequency. First, one has to identify the impact other factors may have on the oscillator precision, that is, to assess the oscillator error unrelated to its supply. Second, the usefulness of the LDO modifications introduced in this work—see Figure 2 and Figure 3 to the LDO reported in [10] should be demonstrated by comparing the impact the two LDOs have on the oscillator error.

Let us evaluate the oscillator accuracy for three supply scenarios, by using the testbench shown in Figure 15: it comprises three instantiations of the oscillator, the first supplied by an ideal voltage source of 1 V, the second supplied by the LDO proposed here—shown in Figure 3—and the third supplied by the LDO reported in [10].

The supply voltages denoted Vout1 and Vout2 in Figure 15 deviate from their nominal value of 1 V due to the LDO offset voltage caused by component mismatches, V_OS_, and the impact of the dynamic loading presented by the oscillator on the LDOs. For this application, one expects the V_OS_ drift with temperature to be particularly important. Therefore, two test scenarios and corresponding measurements were devised to separately analyze the impact the V_OS_ and its thermal drift have on the oscillator accuracy.

**Figure 16:**
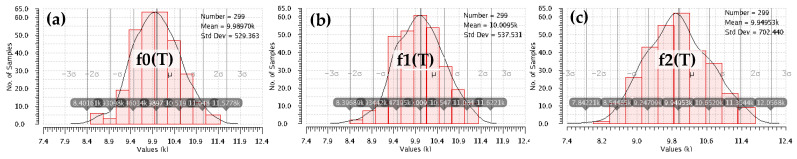
The spread of oscillating frequency caused by component mismatch, considering the three cases for generating the supply voltage for the sensor ring oscillator shown in Figure 15: (**a**) an ideal voltage source, (**b**) the LDO proposed here, shown in Figure 3, and (**c**) the LDO reported in [10]. Results yielded by 300 Monte Carlo simulation runs at +25 °C.

The oscillator frequency error caused by V_OS_ was obtained by running 300 Monte Carlo simulation runs, at T = 25 °C, on the testbench circuit shown in Figure 15. The resulted frequency spread for each supply case is shown side-by-side in Figure 16. The intrinsic error of the oscillator, not related to variations of its supply, can be assessed by analyzing the spread of the frequency obtained for the oscillator supplied by an ideal source, denoted f0(T) in Figure 16a. By comparing it with the corresponding frequency spreads yielded by the same oscillator when supplied by the LDO proposed here—denoted f1(T) in Figure 16b and the one reported in [10] and denoted f2(T) in Figure 16c —one can assess the impact these LDOs have on the oscillator accuracy.

Table 1 lists the parameters that describe the oscillator frequency spread for each of the three supply cases shown in Figure 15. One notices that the oscillator supplied by the proposed LDO exhibits a frequency variation remarkably close to that yielded by the ideal supply case: the standard deviation is only 1.5% larger. The same oscillator supplied by the LDO reported in [10] exhibits a wider frequency spread, resulting in a standard deviation 32% larger than the ideal supply case. These results are in line with the expectations set by simulations presented in Figure 12, which demonstrated that the proposed LDO exhibits a far smaller V_OS_ variation than the LDO reported in [10]. The tighter V_OS_ spread resulted in a 30% narrower spread of the frequency yielded by the oscillator supplied by the proposed LDO, compared with its counterpart in [10].

The relative deviations of the oscillation frequencies f1(T) and f2(T) with respect to the ideal-supply case, f0(T), were computed for each Monte Carlo simulation run. Statistical data on the resulting relative errors are listed in the last two rows of Table 1. This allows one to analyze the error caused by the LDOs separately from the intrinsic error of the oscillator.

The temperature drift of the LDO offset voltage introduces an additional error to the oscillator frequency. This error should be analyzed separately from the one caused by the supply DC shift caused by V_OS_ at room temperature because the latter can be compensated for—at least partially—by one-time trimming performed at room temperature.

The following four-steps simulation procedure was followed:

(1) The V_OS_ variation with temperature of each LDO was monitored over 300 Monte-Carlo runs of DC temperature sweeps.
(2) The worst-case runs that yielded the largest differences between the minimum and maximum V_OS_ values over temperature, were identified.
(3) The corresponding characteristics were shifted by modifying the LDO reference voltage, so that the output voltage reached its nominal value at +25 °C, that is V_OS_ = 0.
(4) The oscillators frequency and error variation with temperature was monitored for each worst-case run identified at step 2.

By using the testbench shown in Figure 15, the intrinsic frequency error of the oscillator, not related to variations of its supply, could be monitored at the same time.

**Figure 17:**
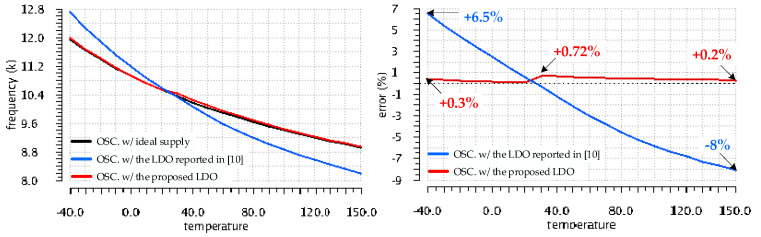
Variation over temperature of the frequency values yielded by the oscillator for the three supply cases shown in Figure 15 and their deviation wrt. the ideal-supply case for the Monte Carlo run that yielded the worst-case V_OS_ temperature drift for the LDO reported in [10].

Figure 17 shows the oscillator frequency and the variation of its error over the full temperature range (from −40 °C to +150 °C) for the Monte Carlo run that yielded the worst-case V_OS_ drift with temperature for the LDO reported in [10]. One notices that the frequency drift with temperature, f2(T), of the oscillator supplied from the LDO reported in [10], is significantly larger than that of the oscillator supplied from an ideal supply, f0(T), yielding an error of up to 8% at +150 °C. By contrast, the frequency, f1(T) of the oscillator supplied by the LDO proposed here exhibits a temperature variation remarkably close to the one supplied by the ideal voltage source: the maximum f1(T) deviation from the ideal case, f0(T), is only 0.72%, registered at +30 °C.

**Figure 18:**
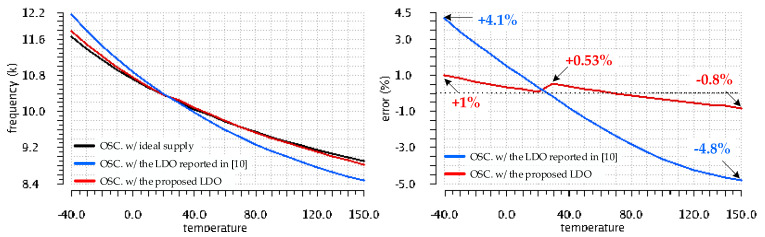
Variation over the temperature of the frequency values yielded by the oscillator for the three supply cases shown in Figure 15 and their deviation wrt. The ideal-supply case for the Monte Carlo run that yielded the worst-case V_OS_ temperature drift for the proposed LDO, shown in Figure 3.

The complementary simulation condition yielded the results shown in Figure 18: the oscillator frequency and the variation of its error over the full temperature range for the Monte Carlo run that yielded the worst-case V_OS_ drift with temperature for the LDO proposed in this work. In this case, f1(T) exhibits a slightly larger temperature variation, with a maximum error of +1.01% at −40 °C. However, even for this worst-case for the proposed LDO, the frequency variation of the oscillator supplied by the LDO reported in [10] is far larger, yielding errors between +4.1% and −4.8%.

Table 2 lists the relative error of frequencies provided by oscillators supplied by the two LDOs compared here, f1(T) and f2(T), with respect to the oscillator supplied by the ideal voltage source, calculated at several temperature points for each of the two Monte Carlo simulation runs. The frequency error of the oscillator supplied by the proposed LDO is up to eight times smaller than the error of the same oscillator supplied by the LDO reported in [10]. These results are in line with the ones presented in Figure 13, which demonstrated that the maximum V_OS_ temperature drift for the proposed LDO is about seven times smaller than for the LDO reported in [10].

### 3.4. Silicon Implementation and Measurement Results

**Figure 19:**
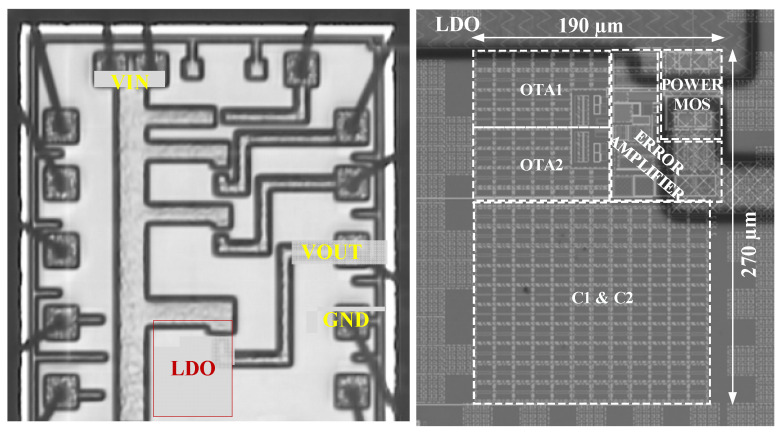
Micrograph of the test-chip section the proposed LDO was integrated in and a zoom-in that provides the LDO floorplan.

The previous section demonstrated that process variations and component mismatches are essential factors that impact the performance of the LDO and oscillator ensemble. It follows that a meaningful experimental validation would require the manufacturing of hundreds of sensor samples on skewed lots and two sets of measurements performed on the integrated sensor and LDO: one should first measure the LDO output voltage and trim out its offset, then measure the sensor accuracy. This is beyond the scope/budget of this research paper. Let us focus instead on measurements performed on the proposed LDO and infer the impact of the integrated LDO performance on the sensor by using the comprehensive analysis presented in Section 3.3. Figure 19 presents the chip micrograph and a zoom-in that details the floorplan of the integrated LDO. About two-thirds of the die area is occupied by the compensation low-density metal capacitors C1 and C2. Using high-density metal capacitors was ruled out for cost reasons.

The measurements presented in this section focus on the features of the LDO described in this work that are essential to its supplying time-domain temperature sensors: fast response to line and load transients and low post-trim output voltage temperature drift.

For measuring the load transient response, we used the test setup detailed in [10], as it is more effective than the one reported in [35]; the charge injection through the parasitic capacitance of the switching transistor is significantly reduced.

**Figure 20:**
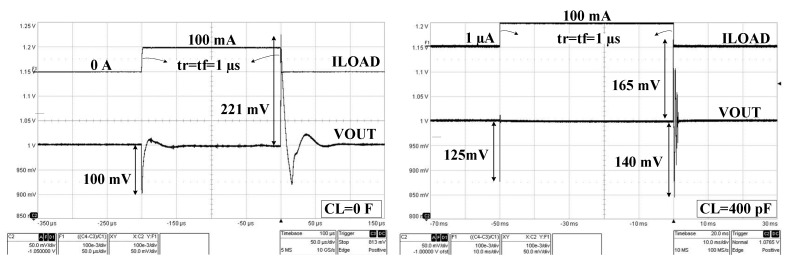
Measured LDO response to a load step between zero and 100 mA in 1 µs for VDD = 1.5 V and two values of the output capacitance: CL = 0 F (**left**) and CL = 400 pF (**right**).

The measured LDO responses to large load current steps, with IL jumping between zero and its maximum value (IL = 100 mA) in 1 µs, for extreme values of the load capacitance, zero and 400 pF, are presented in Figure 20:

- For CL = 0, the LDO response has an undershoot of 100 mV and an overshoot of 221 mV.

- For CL = 400 pF, the output voltage undershoot increases by 25% while the overshoot decreases by 30%. The small ringing present after the overshoot correlates well with the small phase margin shown in Figure 7 for CL = 400 pF.

**Figure 21:**
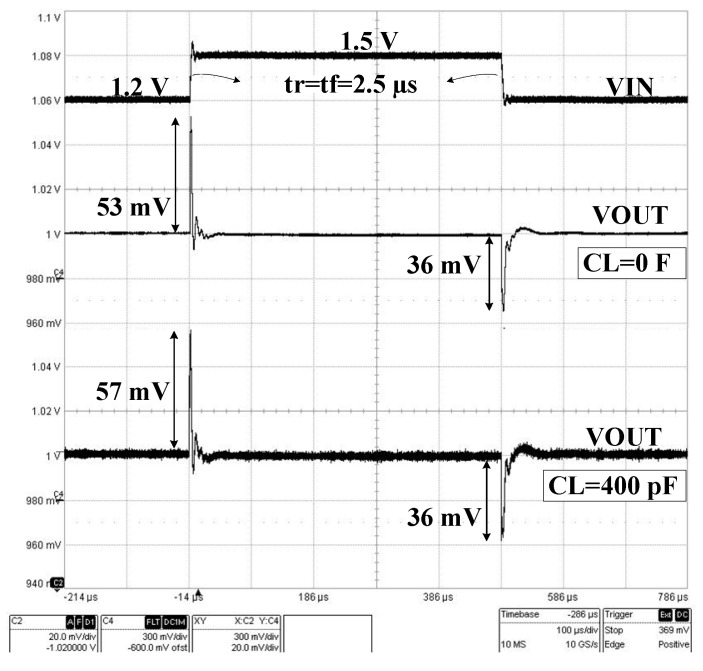
Measured LDO response to a line step for CL = 0 and 400 pF and IL = 1 mA; VIN jumps between 1.2 V and 1.5 V in 2 µs.

Figure 21 presents the measured LDO response to a large and steep line jump, with Vin varying between 1.2 V and 1.5 V in 2.5 µs. For CL = 0, the LDO output voltage has an overshoot of 53 mV and an undershoot of 36 mV; the corresponding values for CL = 400 pF are similar: 57 mV overshoot and 36 mV undershoot.

**Figure 22:**
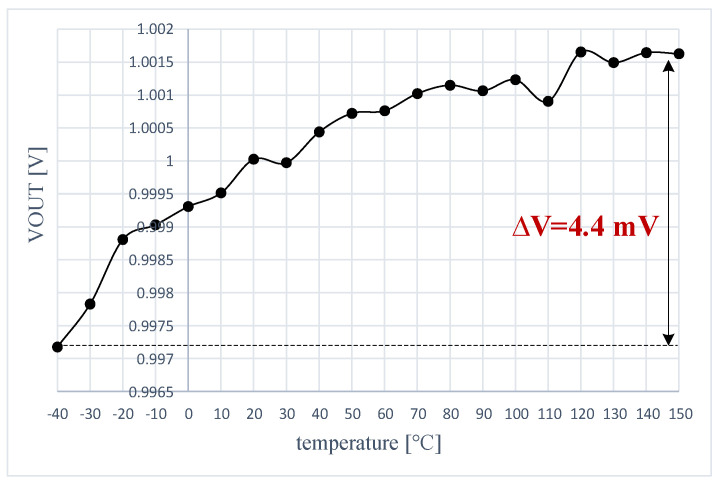
Measured temperature drift of the LDO output voltage error for VIN = 1.5 V, IL = 1 mA, and CL = 100 pF.

The temperature drift of the LDO output voltage error was measured by following the procedure described in Section 3.2, used there to obtain simulation results for the same parameter. Figure 22 presents results yielded by the LDO when supplied at VIN = 1.5 V and loaded by IL = 1 mA and CL = 100 pF. The measured thermal drift of only 4.4 mV over the entire temperature range, from −40 °C to +150 °C, is significantly smaller than the simulated result shown in Figure 13. One notes that measurements yielded better results than simulation. This is explained by the fact that measurements were performed on a test chip for which the process was well controlled, while simulation results considered extreme process corners.

## 4. Comparison with State-of-the-Art and Conclusions

### 4.1. Comparison with State-of-the-Art

Table 3 lists the main parameters of the LDO described here alongside six LDOs reported previously that are able to provide similar output voltages and currents, while operating at similar drop-out voltages. The load capacitors are different—four of the six LDOs can handle only 100 pF, while two can handle larger capacitors than the proposed LDO, 2.2 nF and 1 µF—and so are the quiescent currents and integration processes.

A direct, parameter-by-parameter comparison against the six published LDOs listed in Table 3 indicates that the LDO presented in this work:

(a) Has the second smallest quiescent current, 0.7 µA more than the LDO in [10].
(b) Has the second-largest DC loop gain, 142 dB. This large value improves the LDO performance measured by several parameters of critical importance for high-precision LDOs:
  - First, it helps achieve a very good DC load regulation. The measured load regulation—larger than the simulated value due to voltage drops on the test board tracks—is 1 µ/mA, which is second best to [17].
  - Second, it helps the proposed LDO to achieve a very good supply rejection: the best PSR value at 1 kHz, 20 dB better than second best—the LDOs reported in [17] and [9]—and the second-best PSR value at 10 kHz, only 6 dB less than the LDO in [7].
(c) Is the best in respect to offset voltage: the output voltage error caused by component mismatches, V_OS_, has a standard deviation of σ = 9.5 mV, 3.8 times lower than the LDO reported in [10].

Rather unusually, the LDO presented here exhibits a largely constant unity gain frequency, UGF, of 20 kHz across the entire load current range. In general, such a low UGF results in poor transient performance. However, this is not the case here, as our LDO has the third-best output voltage undershoot, only 26 mV and 6 mV larger than the LDOs reported in [36] and [10], which have far larger UGF—3 MHz and 233 kHz, respectively. Moreover, the total output voltage variation caused by a symmetrical load step, that is the sum of output voltage undershoot and overshoot, is 321 mV for the LDO described here, 40% smaller than the total output voltage variation reported by the LDO in [9], whose UGF goes up to 10 MHz. This is enabled by the LDO architecture presented in Figure 3: a fast error amplifier core that drives the pass transistor by using a local feedback loop, enclosed together with a large gain stage in the main feedback loop, closed between the LDO output and the inverting input of the composite error amplifier. The LDO transient performance is mainly determined by the fast core, while the additional gain stage and the main feedback loop closed around the entire circuit determine the loop gain of the LDO, hence the large DC gain and constant UGF.

Several figures of merit have been introduced to compare the overall performance of LDOs that output similar voltages and currents. The FOM1 was introduced in [37] for comparing regulators designed for different values of quiescent current, $I_{q}$, and load capacitance, CL. Besides these parameters, its definition includes the maximum load current, $I_{L\_max}$, and the output voltage variation caused by load current steps, $\Delta V_{out\_ILstep}$:

(15) $$FOM1=\frac{\Delta V_{out\_ILstep}\cdot C_{L}\;\cdot \;I_{q}}{I_{L\_max}^{2}}$$

A FOM tailored for LDOs designed to operate with no, or very small, load capacitors was introduced in [38]. The slope of the IL step has a decisive impact on the LDO output voltage undershoot/overshoot when there is practically no decoupling capacitance.

**Table 3:** Performance Comparison.

Parameter	[36] ^†^	[6] ^†^	[19] ^††^	[9] ^†^	[35] ^†^	[10] ^†^	This Work
Year	2010	2012	2016	2019	2020	2020	**2021**
CMOS [μm]	0.35	0.35	0.5	0.065	0.065	0.13	**0.13**
FO4Delay(ps) ^(c)^	90	90	130	17	17	35	**35**
Supply Voltage [V]	2.4–3.3	2.5–4	2.3–5.5	0.95–1.2	0.95–1.2	1.2–1.5	**1.25–1.5**
CL [F]	100 p	0–100 p	0–2.2 n	0–100 p	0–100 p	0–1μ	**0–400 p**
DC							
Output Voltage [V]	2.2	2.3	1.2–5.4	0.8	0.8	1	**1**
Output current range (IL_MIN_–IL_MAX_)	0–100 mA	50μ–100 mA	0–150 mA	0–100 mA	0–100 mA	0–100 mA	**0–100 mA**
Iq [μA]	31	7	40	13.9	14	0.7	**1.4**
Dropout Voltage [mV]	200	150	100	150	150	100	**150**
DC line reg. [mV/V]	623	1	0.028 ^(e)^	0.48	12	16.6	**3.3**
DC load reg. [μV/mA]	2.31	80	0.5 ^(e)^	8.03	90	100	**1**
V_OS_ (3 σ) @ room temp	–	–	–	–	–	105 mV *	28.8 mV *
V_OS_ thermal drift (post-trim)	–	–	–	–	–	+83.3 mV * −91.4 mV	+12 mV * −14.4 mV *
AC * and STB *							
Loop gain @ DC [dB]	–	–	159	62	71	80	**142**
Min. phase margin @ CL = 0 and room temp	–	–	83°	41°	52° ^(b)^	10°	**40**°
UGF [Hz] @ IL = 0 A	–	–	2 M	1 M	0.66 M ^(b)^	72 k	**20 k**
UGF [Hz] @ IL = ILmax	–	–	3 M	10 M	9 M ^(b)^	233 k	**20 k**
PSR [dB]	60@1 kHz * 40@10 kHz *	–	65@1 kHz * ^(a)^ 65@10 kHz * ^(a)^	65@1 kHz * ^(a)^ 47@10 kHz *	34@1 kHz * 33@10 kHz *	50@1 kHz * 30@10 kHz *	85@1 kHz * 41@10 kHz *
Response to load steps							
Load step							
IL_MIN_–IL_MAX_//Avg. IL t_rise_	0–100 mA/1000 ns	50 μ–100 mA/500 ns	0–150 mA/1000 ns	0–100 mA/50 ns	0–100 mA/132.5 ns	0–100 mA/1000 ns	**0–100 mA/1000 ns**
Rise time ratio (K)	20	10	20	1	2.65	20	**20**
Undershoot [mV]	65.1	236 ^(b)^	106	404	230	76	**100 ^(d)^**
Overshoot [mV]	67	227 ^(b)^	115	145	133	198	**221 ^(d)^**
$\Delta V_{out\_ILstep}$ [mV] (Undershoot + Overshoot)	132	463	221	549	363	274	**321**
**FOM1 [fs]**	40.95	33.03	3.93 **	7.63 **	50.82	0.19 **	**0.45** **
**FOM2 [mV]**	0.82	0.33	1.18	0.08	0.13	0.04	**0.09**
**FOM3 [V/μs]**	1.24	6.38	1.23	4.49	4.14	0.15	**0.35**

^(a)^ Estimated from figure; ^(b)^ for CL = 100 pF; ^(c)^ values taken from [39]; ^(d)^ at CL = 0 pF; ^(e)^ computed for VOUT = 1.2 V; * from simulation; ** a CL value of 10 pF was considered instead of 0 F; ^†^ designed for SoC applications; and ^††^ designed for mobile applications. Bold here highlighted the performance of the circuit proposed in this paper as opposed to the performance reported in previous published papers.

Therefore, instead of the CL value—presumably similarly small for such LDOs—the rise/fall time of the load current step is taken into account, albeit in an indirect manner:

(16) $$FOM2=K\frac{\Delta V_{out\_ILstep}\;\cdot \;I_{q}}{\Delta I_{L}}$$

in which $K=\frac{\Delta t\;used\;in\;measurement}{the\;smallest\;\Delta t\;among\;desings\;for\;comparison}$.

An expanded version of the FOM2 defined by (16) was introduced in [39] to take into account two other factors that impact the LDO response to load steps: (i) the minimum load currents the LDO can handle, or the minimum IL value used for the load step; and (ii) the propagation delay estimated of an inverter with the fan-out of four, FO4, as an indicator of the speed-related performance of the process the LDO is integrated in:

(17) $$FOM3=K^{1/3}\left[\frac{\Delta V_{out\_ILstep}\;\cdot \;\left(I_{q}+I_{L\_min}\right)}{FO4_{Delay}\cdot \Delta I_{L}}\right]$$

For all these FOMs, the smaller the value, the better the LDO transient performance.

The LDO presented in this work is the second best with respect to FOM1 and FOM3. Its FOM2 value is very close to the second best, 0.08, yielded by the LDO reported in [9]. One might conclude that the overall performance of our LDO is second best to the LDO in [10]. However, the LDO presented here was optimized for supply-sensitive time-domain temperature sensors. In this respect, it is far better than the LDO reported in [10]:

- Its output voltage error caused by component mismatches and variations of the line voltage, load current, and temperature is less than +/−35 mV. The LDO reported in [10] exhibits a DC offset of about 100 mV, with a temperature drift over 150 mV;
- The thermal drift of the output voltage offset caused by component mismatches, across the temperature range of −40 °C to +150 °C, is 9.5 smaller for our LDO than the one provided by the LDO in [10], which was integrated in the same process.

### 4.2. Conclusions

The LDO topology presented in this work is based on multiple feedback loops: the core loop encompasses the fast error amplifier that drives the pass transistor, which has its response to load and line transients further augmented by a local feedback loop; additional gain is provided by a two-stage composite OTA, which is enclosed together with the core loop in the main feedback loop, closed between the LDO output and the inverting input of the composite amplifier. The LDO transient performance is mainly determined by the fast core and its local feedback, while the additional gain stage within the main feedback loop ensures excellent load regulation and PSR. Moreover, the composite OTA can be optimized for low offset and temperature drift, without impacting the transient response. A novel approach to frequency compensation allows the LDO to maintain a large DC gain while handling a wide range of load currents and capacitors. This avoids the trade-off between fast transient response, large DC gain, and stability for various loading conditions that limit the performance of conventional LDOs.

The proposed topology was used to implement a high-precision LDO with fast response to load transients, suitable for supply-sensitive time-domain temperature sensors.

Simulation and measurement results performed on a test chip implemented in standard 130 nm CMOS process validated the proposed LDO. The LDO requires only 1.4 µA quiescent current but exhibits an excellent response to load transients. When the load current jumps from 0 A to 100 mA in 1 µs, the output voltage presents an undershoot of 100 mV and an overshoot of 221 mV, without decoupling capacitors. Due to its high DC gain, the LDO exhibits very good DC load regulation: 1 µV/mA. Moreover, the output-referred LDO offset voltage exhibits a small spread (σ = 9.5 mV) and a very low thermal drift: 14.4 mV over the temperature range of −40 °C to +150 °C, across process variations and mismatch. Overall, the output voltage error caused by component mismatches and variations of the line voltage, load current, and temperature does not exceed +/−35 mV.

The performance of the LDO described here was compared against six published LDOs designed for similar levels of supply voltage and output voltage and current. Our proposal came first for PSR at 1 kHz, delivering 20 dB more than second best. The proposed LDO also delivered the smallest thermal drift of the output offset, 6.7 times lower than its counterpart.

A comprehensive set of simulations were presented for the ensemble LDO and temperature-dependent ring oscillator at the core of the sensor. It supported a comparative analysis of the impact the supply sources have on the oscillating frequency over the automotive temperature range. The smaller offset voltage, with a smaller temperature drift, exhibited by the proposed LDO resulted in a significant reduction in the frequency error registered for the oscillator it supplied, compared with the error registered for the same oscillator when supplied by a general purpose LDO reported earlier. Over the temperature range of −40 °C to +150 °C, the respective errors took the following values: from +1% to −0.8%, compared with +4.1% to −4.8%. 

## Figures and Tables

**Table 1:** First three rows: parameters that describe the oscillator frequency spread for each of the three supply cases shown in Figure 15. Last two rows: relative deviations of the oscillation frequencies f1(T) and f2(T) with respect to the ideal-supply case, f0(T). Results of 300 Monte Carlo runs at +25 °C.

T = 25 °C	Min	Mean	Max	Std Dev
f0(T) [kHz]	8.49	9.99	11.4	0.5294
f1(T) [kHz]	8.42	10	11.3	0.5375
f2(T) [kHz]	8.03	9.95	11.7	0.7024
f1(T) error wrt f0(T) [%]	−4.414	0.2	3.199	1.273
f2(T) error wrt f0(T) [%]	−15.36	−0.39	12.16	4.79

**Table 2:** Deviation from the ideal-supply case of frequencies provided by oscillators supplied by the LDO reported in [10] and the LDO proposed here, for the Monte Carlo simulation runs that yielded the worst-case V_OS_ temperature drift for each LDO.

T [°C]	Worst Case V_OS_ Temp. Drift for the LDO Reported in [10]		Worst Case V_OS_ Temp. Drift for the Proposed LDO	
	Error f1(T) [%]	Error f2(T) [%]	Error f1(T) [%]	Error f2(T) [%]
−40	0.34	6.50	1.01	4.15
−20	0.25	4.40	0.66	2.79
0	0.15	2.47	0.36	1.54
20	0.04	0.62	0.08	0.36
40	0.66	−1.23	0.39	−0.76
60	0.53	−2.99	0.13	−1.87
80	0.45	−4.58	−0.10	−2.85
100	0.40	−5.85	−0.32	−3.63
120	0.35	−6.85	−0.51	−4.24
140	0.30	−7.69	−0.71	−4.69
150	0.25	−8.07	−0.82	−4.84
